# NKG2D CAR-T cells for solid tumor immunotherapy: advances, challenges, and future directions

**DOI:** 10.3389/fimmu.2026.1763843

**Published:** 2026-02-11

**Authors:** Chen Liu, Zhiqiang Wang, Wentao Zhang, Guangping Cheng, Siyan Cheng, Long Qin, Huili Ye, Wen Ren

**Affiliations:** 1 Cuiying Biomedical Research Center, The Second Hospital & Clinical Medical School, Lanzhou University, Lanzhou, Gansu, China; 2 Department of Stomatology, The Second Hospital & Clinical Medical School, Lanzhou University, Lanzhou, Gansu, China; 3 Department of General Surgery, The Second Hospital & Clinical Medical School, Lanzhou University, Lanzhou, Gansu, China; 4 Department of General Practice, The Second Hospital & Clinical Medical School, Lanzhou University, Lanzhou, Gansu, China; 5 Gansu Tumor Immunology Basic Disciplines Research Center, The Second Hospital & Clinical Medical School, Lanzhou University, Lanzhou, Gansu, China

**Keywords:** combination therapy, engineering design, NKG2D CAR-T cells, solid tumors, TME

## Abstract

Chimeric antigen receptor (CAR) T-cell therapy has achieved significant success in hematologic malignancies, but its efficacy in solid tumors remains limited, primarily due to the immunosuppressive tumor microenvironment (TME) that hinders CAR-T cell trafficking and function. NKG2D CAR-T cells, which target stress-induced NKG2D ligands (NKG2DLs) broadly expressed on tumor cells, have shown promising potential in overcoming the immunosuppressive barriers of the solid TME. This review highlights recent advances in NKG2D CAR-T cell strategies for solid tumors, including innovations in CAR architecture, signaling pathway engineering, combination immunotherapy, and the development of armored CAR constructs. We further discuss the therapeutic potential, current challenges, and future directions of these approaches to inform the design of more effective and durable CAR-T cell therapies for solid tumors.

## 1 Introduction

CAR-T cell therapy has demonstrated remarkable efficacy in the treatment of hematological malignancies, yielding remarkable clinical responses in leukemia, multiple myeloma, and B-cell lymphomas, but its therapeutic efficacy in solid tumors remains limited (1, 2). First, the high heterogeneity of solid tumors and the diversity of tumor-associated antigens (TAAs) present significant challenges, particularly antigen escape and off-target effects that can result in unintended attacks on healthy tissues and cells (3). Second, the immunosuppressive TME within solid tumors significantly hinders CAR-T cell infiltration and function through mechanisms such as chronic inflammation, accumulation of immunosuppressive cells, and aberrant vasculature (4, 5). Although CAR-T cell therapy faces numerous challenges in solid tumors, research is progressively overcoming these limitations through multifaceted strategies including target optimization, CAR structural engineering, TME remodeling, combination immunotherapy, and innovative delivery strategies (6–8).

NKG2D is a key activation receptor in the innate immune system that recognizes multiple stress-induced ligands, known as NKG2DLs, including MICA and MICB, and the ULBP1-6, which are frequently overexpressed in cancer cells (9, 10). In solid tumors, cancer cells upregulate the expression of NKG2DLs in response to cellular stress, DNA damage, dysregulated proliferation, and other processes, making the NKG2D/NKG2DL axis a promising target for immunotherapy (11–14).However, cancer cells can evade immune surveillance by secreting soluble NKG2DLs, downregulating surface ligand expression, or remodeling the TME to hinder immune cell infiltration and impair their function. These immune evasion mechanisms present major challenges to the development and efficacy of NKG2D CAR-T cell therapy in solid tumors (9, 10, 14–16).

This review provides a comprehensive analysis of the advantages and limitations of NKG2D CAR-T cell therapy in the context of solid tumor immunotherapy, with a particular focus on its potential to overcome the immunosuppressive TME. We summarize key strategies employed to counteract tumor immune evasion and suppression, highlight recent preclinical advances, and examine the heterogeneity in treatment responses as well as organ-specific challenges observed across various solid tumor types. Finally, we explore future directions and combinatorial approaches for enhancing NKG2D CAR-T cell efficacy, emphasizing strategies aimed at remodeling the TME to improve CAR-T cell infiltration, persistence, and antitumor activity. These insights are expected to provide a theoretical foundation and practical guidance for the translational application of NKG2D CAR-T cell therapy in the future.

## 2 NKG2D and its ligands

### 2.1 Structural architecture of NKG2D

NKG2D (Natural Killer Group 2D) is a key activating receptor expressed on human and murine NK cells, CD8^+^ T cells, and subsets of other immune effector cells, but is typically absent on CD4^+^ T cells (9, 16). NKG2D, encoded by the KLRK1 gene, is a 42 kDa type II transmembrane receptor (17). The extracellular region of NKG2D contains a C-type lectin-like domain that recognizes multiple stress-induced ligand proteins. Signal transduction is mediated through the binding of its cytoplasmic domain to DNAX-activating protein 10 (DAP10), enabling activation signaling (15). The cytolytic activity of NKG2D is dependent on DAP10 homodimers bound to its cytoplasmic domain. This complex activates cells through downstream PI3K and Grb2 signaling pathways, directly triggering NK cell cytotoxicity. Additionally, NKG2D acts as a co-stimulatory signal to potentiate the activation and enhance the cytotoxic function of αβ T cells and γδ T cells.

### 2.2 NKG2DLs

**Figure 1:**
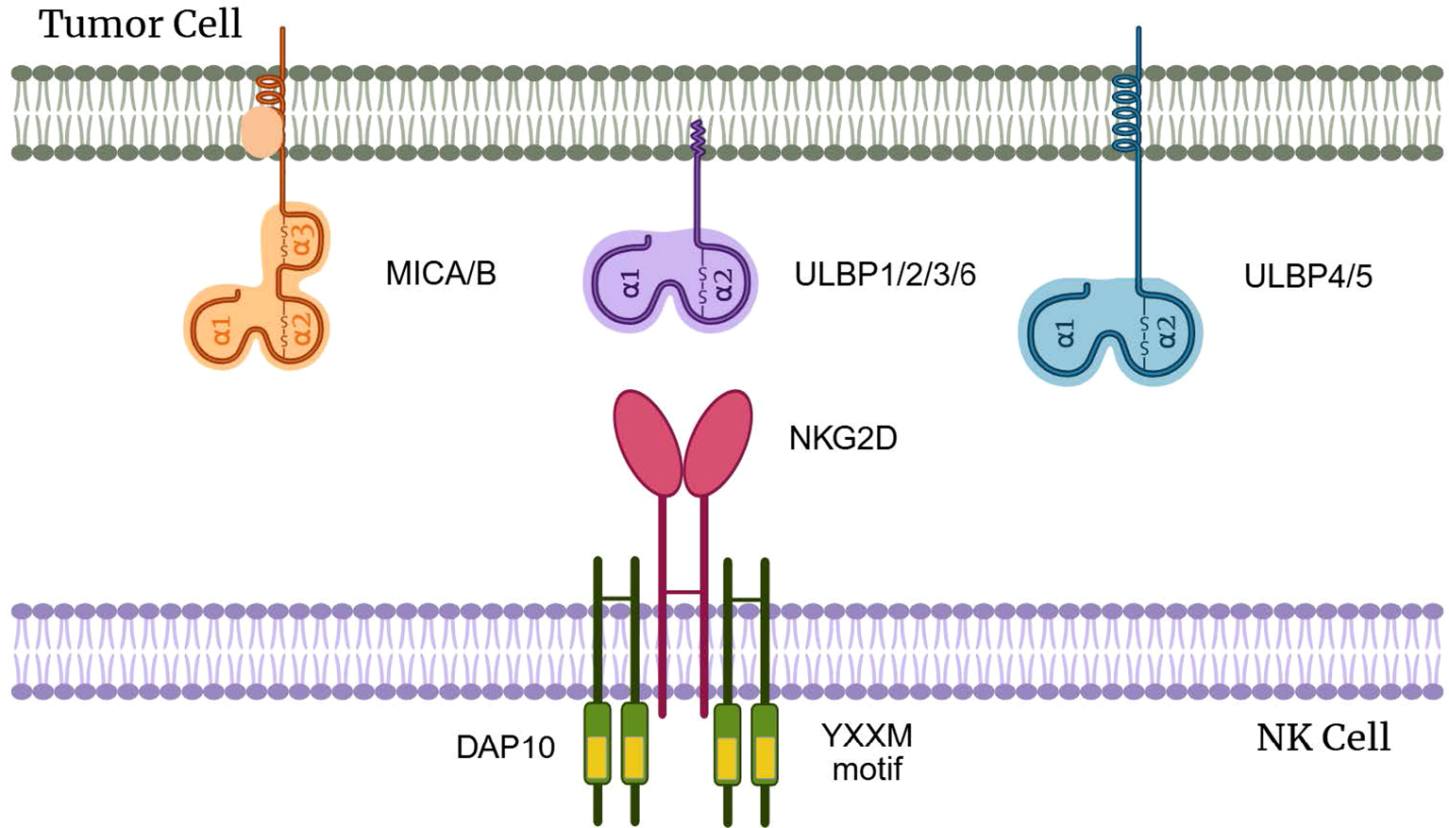
The structure of NKG2D and NKG2DLs. This graphical abstract was created with BioRender.com (License *IE28PZFH3A*).

NKG2DLs include the human MHC class I chain-related molecules (MICA and MICB) and members of the UL16-binding protein (ULBP) family (ULBP1-6) (9, 10). These ligands are minimally expressed in normal tissues but are strongly upregulated in response to cellular stress, including malignant transformation and viral infection. For instance, MICA and MICB are expressed in 100% of colorectal tumors, 97% of breast cancers, 95% of renal cell carcinomas, 81% of ovarian cancers, 77% of primary cutaneous melanomas, and 50% of primary uveal melanomas (18–22). MICA and MICB belong to the MHC class I molecule family but differ from classical MHC class I molecules in that they do not associate with β_2_-microglobulin (23–27). ULBPs (ULBP1, ULBP2 and ULBP3 are either glycosylphosphatidylinositol (GPI)-anchored or transmembrane proteins. They share structural domains similar to MICA/MICB but lack the immunoglobulin-like fold characteristic of MHC class I molecules (28–34) (**Figure 1**).

### 2.3 Signaling transduction of the NKG2D pathway

Upon ligand engagement, NKG2D associates with the adaptor DAP10 via its transmembrane domain to form a dimeric signaling complex, which in turn activates downstream PI3K/Akt and Grb2/Vav1 signaling pathways (15, 17). These cascades promote cytotoxic immune responses by enhancing cytokine secretion (e.g., IFN-γ) and granzyme B release, leading to apoptosis of tumor or stressed cells. In addition to its direct effector function, NKG2D also provides co-stimulatory signals that augment T cell activation. Within the TME, NKG2D synergizes with other activating receptors to amplify anti-tumor immunity.

### 2.4 NKG2D/NKG2DL in tumor immunity

**Figure 2:**
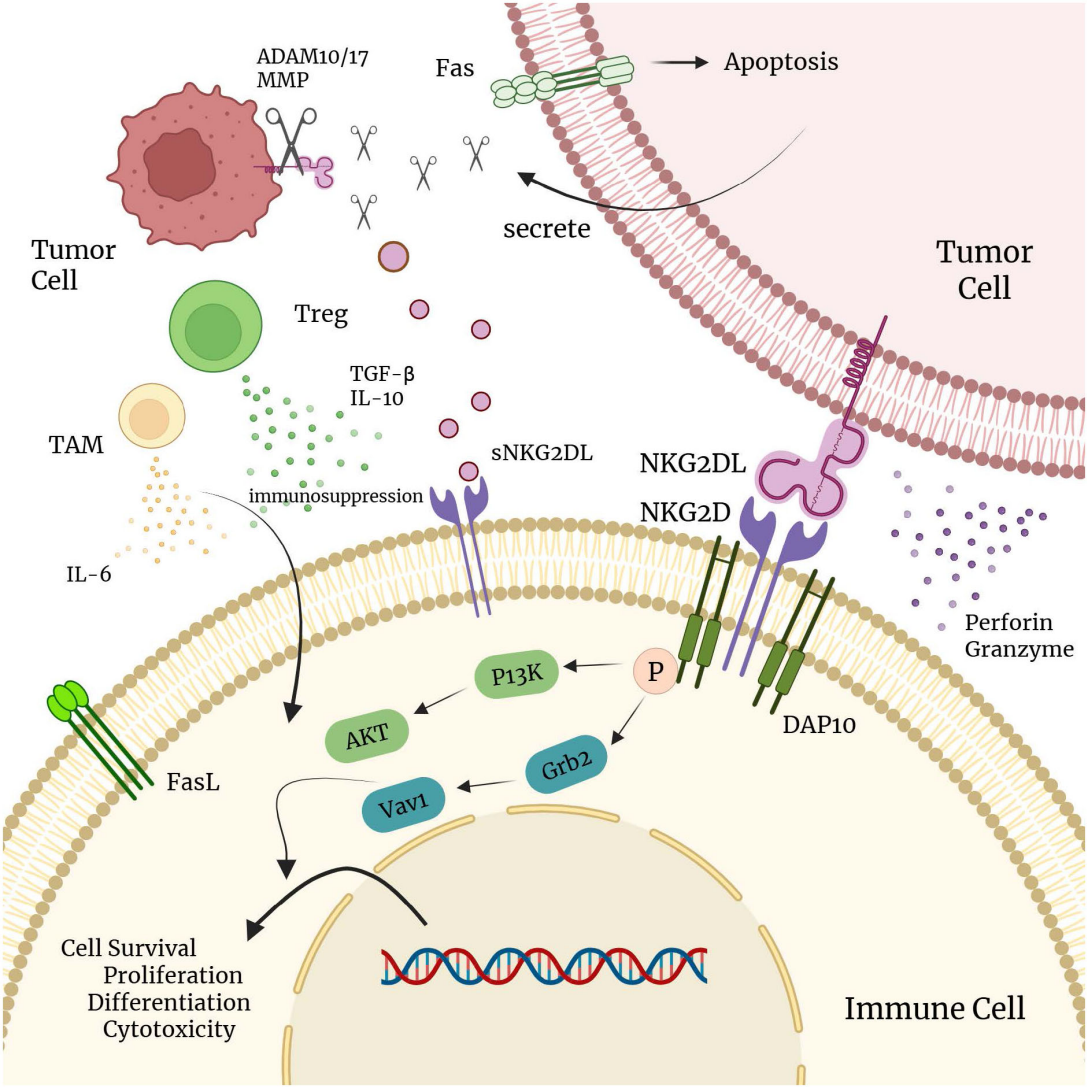
The role of NKG2D in tumor immunity. This graphical abstract was created with BioRender.com (License *IZ28PZFVVM*).

NKG2D-mediated recognition of its ligands plays a critical role in tumor immune surveillance (13). In early tumorigenesis, malignant cells often upregulate NKG2DLs to recruit NK cells and CD8^+^ T cells for tumor elimination. However, tumor cells evolve mechanisms to evade NKG2D-driven immune pressure (10, 35). For instance, tumor cells can upregulate proteases such as ADAMs and MMPs to cleave membrane-bound NKG2DLs (e.g., MICA/B), releasing soluble forms that bind to NKG2D receptors on immune cells, induce receptor internalization and downregulation, and ultimately suppress NK and CD8^+^ T cell activation to promote immune escape (36–39). Furthermore, immunosuppressive factors and cells within the TME remodel the immune landscape: inhibitory cytokines such as TGF-β downregulate NKG2DL/NKG2D expression on both tumor and immune cells, while activation of immune checkpoint pathways like PD-1/PD-L1 induces T cell exhaustion, thereby diminishing CAR-T cell cytotoxicity (40–44). Regulatory T cells (Tregs), myeloid-derived suppressor cells (MDSCs), hypoxia, and metabolic constraints within the TME also impede the infiltration and function of immune effector cells (45, 46). Additionally, tumor cells can epigenetically silence NKG2DL expression. For instance, histone H3K27 trimethylation (H3K27me3) leads to transcriptional silencing of NKG2DL genes (e.g., ULBP2). Concurrently, post-translational modifications such as ligand glycosylation and endoplasmic reticulum (ER) stress pathways also impair the maturation and surface expression of NKG2DL proteins (35, 47). Collectively, these multifaceted mechanisms contribute to tumor immune escape from NKG2D-mediated immune surveillance (**Figure 2**).

## 3 CAR and NKG2D CAR

CARs are synthetic receptors designed to redirect immune effector cells, such as T cells or natural killer (NK) cells, to recognize TAAs in an MHC-independent manner. By leveraging the fundamental role of NKG2D in innate immune surveillance, CAR technologies have been developed to exploit analogous activation pathways, thereby augmenting antitumor responses. This section begins by tracing the structural and functional evolution of CAR designs-from first- to fifth-generation constructs-with emphasis on critical advances in signaling efficacy, persistence, and safety profiles. It subsequently details the design rationale for NKG2D-based CARs, which capitalize on the high expression of NKG2DLs on tumor cells, and summarizes unique engineering strategies reported in recent studies.

### 3.1 Evolution of CAR structures: from first to fifth generation

**Figure 3:**
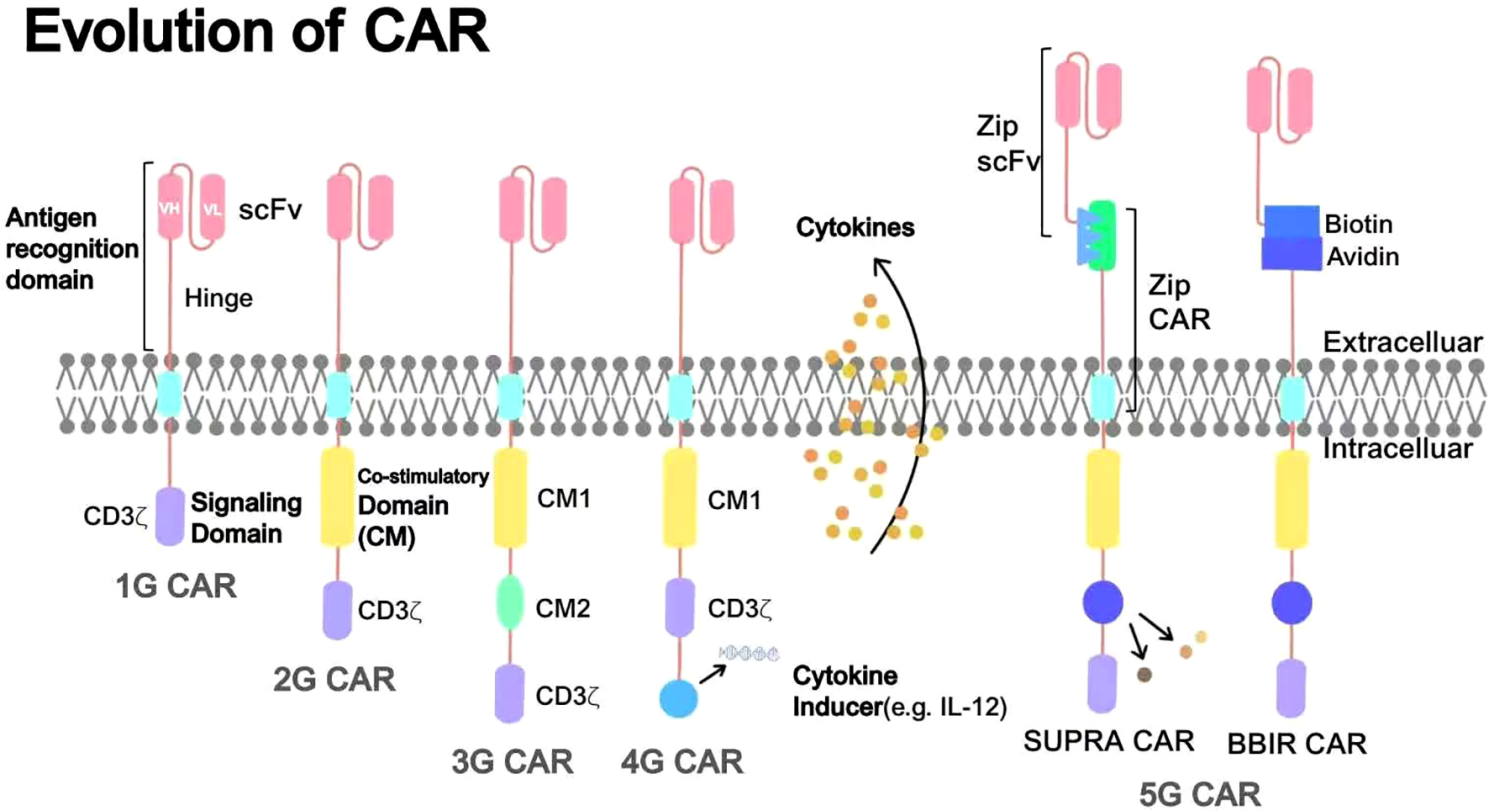
Evolution of CAR structures.

The evolution of CARs has progressed through several generations, with each iteration introducing structural refinements to enhance T-cell activation, proliferation, persistence, and antitumor efficacy, while addressing limitations such as insufficient signaling and CRS (**Figure 3**). Initial CAR designs focused on enabling major histocompatibility complex (MHC)-independent antigen recognition, evolving from simple antibody-based constructs to sophisticated multidomain receptors that incorporate co-stimulatory and cytokine signaling components (48).

First-generation CARs (1G CARs), initially developed in the late 1980s and early 1990s, were designed with an extracellular antigen-binding domain-usually a single-chain variable fragment (scFv) derived from a monoclonal antibody-linked to a transmembrane domain and an intracellular signaling domain, most frequently the CD3ζ chain from the T-cell receptor (TCR) complex. This architecture permitted MHC-independent antigen recognition and T-cell-mediated cytotoxicity, but it provided only the primary activation signal (Signal 1) and lacked co-stimulatory signaling. As a result, 1G CAR-T cells exhibited restricted proliferative capacity, shortened persistence *in vivo*, and insufficient long-term antitumor activity. Although preclinical studies indicated modest antitumor effects, subsequent clinical trials revealed rapid T-cell anergy and limited therapeutic efficacy, underscoring the necessity of integrating co-stimulatory elements in subsequent CAR designs (49).

Second-generation (2G) CARs introduced a co-stimulatory domain, such as CD28 or 4-1BB (CD137), alongside CD3ζ, to mimic the dual-signal requirement for full T cell activation. CD28-based constructs promote rapid proliferation and IL-2 secretion, enhancing short-term effector functions, while 4-1BB favors memory T cell formation and long-term persistence through anti-apoptotic pathways like Bcl-2 upregulation. This generation marked a breakthrough, with CD19-targeted 2G CAR-T cells achieving FDA approvals for B-cell malignancies, including Kymriah^®^(tisagenlecleucel)and axicabtagene ciloleucel (50, 51). Nevertheless, several challenges remain, particularly in the treatment of solid tumors. These include heterogeneous therapeutic responses attributable to the highly immunosuppressive TME, as well as variable *in vivo* persistence of CAR-T cells, which is significantly influenced by the selection of the co-stimulatory domain.

Third-generation (3G) CARs built on 2G designs by incorporating two co-stimulatory domains (e.g., CD28 and 4-1BB or OX40) with CD3ζ, aiming to amplify signaling for superior proliferation and cytokine production. This configuration enhances PI3K/Akt and NF-κB pathways, improving effector functions and resistance to exhaustion. Preclinical models showed increased antitumor potency compared to 2G CARs, particularly in low-antigen-density tumors, but clinical data remain mixed, with some studies reporting no significant persistence gains and heightened risks of CRS due to overactivation (52).

Fourth-generation (4G) CARs, often termed “armored” or TRUCK (T cells redirected for universal cytokine-mediated killing) CARs, integrate inducible cytokine expression (e.g., IL-12, IL-15, or IL-18) under NFAT-responsive promoters, enabling localized cytokine release upon antigen engagement. This design remodels the TME by recruiting endogenous immune cells, countering immunosuppression, and boosting CAR-T persistence through autocrine/paracrine signaling. For instance, IL-7-secreting 4G CAR have demonstrated enhanced infiltration and efficacy in prostate tumor models, though safety concerns like cytokine storms necessitate suicide gene incorporation (53).

Ordinarily, CAR-T cell therapy involves the ex vivo engineering of a patient’s autologous T cells followed by their infusion back into the body. As a highly personalized treatment, each therapy is tailored to an individual patient, resulting in extremely high treatment costs. Consequently, in the development of fifth-generation (5G) CAR-T technology, researchers have focused on overcoming individual limitations, achieving large-scale production and treatment, and reducing costs. Universal CARs utilize two “off-the-shelf” systems-BBIR CAR (Biotin-Binding Immune Receptor) and SUPRA CAR (Split, Universal, and Programmable)-which separate the extracellular antigen-targeting domain from the T cell signaling unit to endow CAR-T cells with the ability to recognize multiple antigens. Meanwhile, T cells obtained from allogeneic healthy donors can be genetically edited ex vivo using technologies such as ZFN, TALEN, and CRISPR/Cas9 to disrupt the TCR and HLA class I genes in T cells, thereby eliminating graft-versus-host disease (GVHD). Addressing this challenge requires overcoming numerous barriers; however, its substantial economic and social benefits have attracted many researchers, and significant progress has increasingly been made (54).

The progressive refinement of CAR structures through successive generations has been a key driver in the evolution of CAR-T cell therapies, significantly advancing the field of cancer treatment. First-generation CAR-T cells demonstrated limited antitumor activity due to the absence of co-stimulatory signaling, resulting in poor persistence and transient responses. In contrast, while third- and fourth-generation CARs exhibit enhanced effector functions and the ability to modulate the TME, they are also associated with increased risks of toxicity, such as CRS and on-target/off-tumor effects. Owing to a more favorable efficacy–safety profile, second-generation CAR-T cells—which incorporate a single co-stimulatory domain such as CD28 or 4-1BB remain the cornerstone of current clinical practice. To date, multiple second-generation CAR-T products have received FDA approval, with their numbers increasing annually, underscoring their leading role in the cellular immunotherapy landscape. Continued progress in preclinical and early-phase clinical trials suggests that CAR-T-based approaches are continuing to develop at a rapid pace.

### 3.2 NKG2D-based CAR designs

NKG2D-based CARs have developed alongside conventional scFv-driven CAR generations, leveraging the natural NKG2D/NKG2DL axis for immune activation. Unlike scFv-CARs, which typically target a single tumor-associated antigen, NKG2D CARs inherently recognize a broad repertoire of stress-induced ligands-including MICA, MICB, and ULBP1-6-that are frequently upregulated on malignant cells but generally low or inducible expression in normal tissues. This multi-ligand targeting capability enables a versatile antitumor response, effectively countering antigenic heterogeneity, a key limitation in the immunotherapy of solid tumors. To reconcile broad target coverage with ligand specificity, their molecular architecture has been progressively refined, integrating design elements from first- to fifth-generation CARs while adopting either pan-ligand or single-ligand targeting strategies tailored to specific therapeutic contexts.

Early NKG2D CAR designs aligned with first-generation (1G) conventional CARs, consisting of full-length NKG2D (amino acids 1-216) or its extracellular domain (ECD, amino acids 73-216) fused to the CD3ζ signaling domain. This minimalist structure relied on endogenous DAP10 association to activate PI3K/Akt and Grb2/Vav1 pathways, enabling recognition of all NKG2DLs and triggering CAR-T cell cytotoxicity and IFN-γ secretion. Preclinical studies in ovarian cancer models demonstrated potent tumor eradication, as these CAR-T cells effectively targeted bulk tumor cells with elevated NKG2DL expression (55). However, similar to 1G CARs, the lack of co-stimulatory signaling led to limited *in vivo* persistence, while ligand-induced NKG2D downregulation and fratricide (due to endogenous NKG2DL expression on activated T cells) constrained therapeutic efficacy. To address these issues, early optimizations included shRNA-mediated silencing of MICA/B in CAR-T cells (e.g., CYAD-02 construct), which reduced self-recognition and improved *in vivo* survival and antitumor activity in glioblastoma xenografts (56).

Subsequent iterations of NKG2D CARs mirrored second-generation (2G) and third-generation (3G) conventional CARs, integrating one or two co-stimulatory domains to enhance activation, persistence, and resistance to exhaustion. The most widely adopted design incorporates the NKG2D ECD fused to 4-1BB/CD3ζ or CD28/CD3ζ signaling modules. 4-1BB-containing NKG2D CAR-T cells favor central memory T cell differentiation, promoting long-term persistence and milder CRS, as observed in preclinical models of HCC-where these cells maintained stable CAR expression, enhanced cytokine secretion, and sustained tumor suppression compared to CD3ζ-only constructs (57).

A key advancement in mitigating the “on-target/off-tumor” toxicity risk inherent to the broad, pan-ligand recognition of natural NKG2D is the development of CARs engineered to target single NKG2DLs. This approach capitalizes on the distinct expression profiles of individual ligands (e.g., MICA, MICB, ULBP1-6) across various tumor types, thereby improving tumor selectivity and reducing potential on-target toxicity against non-malignant stressed tissues which may express a different repertoire of ligands. For example, ULBP2-specific CAR-T cells-engineered by replacing the NKG2D ECD with an anti-ULBP2 scFv-have demonstrated dual efficacy in gastric cancer models: they not only eliminate ULBP2-expressing tumor cells but also remodel the immunosuppressive TME by inhibiting cancer-associated fibroblast (CAF) activation and collagen deposition (58). This single-ligand targeting strategy mitigates the risk of cross-reactivity with healthy cells expressing low levels of NKG2DLs, while addressing antigen escape caused by heterogeneous ligand expression in tumors. NKG2D-CAR structures capable of secreting specific cytokines have been increasingly reported in research. Specifically, proteins that can activate cytokine expression pathways are linked to the C-terminus of the NKG2D receptor protein, thereby endowing NKG2D-CAR with unique functions.

Despite considerable progress, NKG2D-based CAR-T cell therapy continues to face several persistent challenges. These include receptor downregulation induced by soluble NKG2DLs (e.g., MICA/B), profound immunosuppression within the TME-mediated by factors such as TGF-β and MDSCs and heterogeneous expression of NKG2DLs across different tumor regions. Clinical evaluations, such as the phase 1 THINK trial investigating CYAD-01 (an autologous NKG2D CAR-T product), have demonstrated a manageable safety profile but also revealed variable antitumor responses and limited persistence of CAR-T cells in patients, which has been partly attributed to the presence of soluble MICA in patient sera. Current optimization strategies are increasingly focused on combining NKG2D CAR-T cells with TME-modulating agents-for instance, matrix metalloproteinase (MMP) inhibitors to mitigate ligand shedding, or immune checkpoint blockers (e.g., anti-PD-1) to alleviate T-cell exhaustion. Additionally, the development of dual-targeting CAR designs (e.g., NKG2D conjugated with CLDN18.2-specific CARs for gastric cancer) aims to overcome antigen heterogeneity while simultaneously countering TME-driven suppression.

In summary, the architectural evolution of NKG2D CARs has progressed in parallel with conventional CAR technologies, advancing from minimal first-generation designs to sophisticated third- and fourth-generation platforms incorporating multiple co-stimulatory domains and immunomodulatory components. By strategically integrating enhanced co-stimulation, single-ligand targeting precision, and TME-remodeling capabilities, these next-generation NKG2D CAR-T cells more effectively exploit the endogenous NKG2D/NKG2DL axis to target antigenically heterogeneous solid tumors while mitigating previous limitations. Future refinements will likely emphasize precision control over ligand recognition, further optimization of co-stimulatory and cytokine signaling architectures, and rationally designed combination regimens to fully realize the translational potential of NKG2D-directed therapies in solid tumor immunotherapy.

## 4 Preclinical advances of NKG2D CAR-T cells in solid tumors

To date, substantial preclinical research has focused on evaluating NKG2D-based CAR-T cell therapy across a wide spectrum of solid tumors, such as brain tumors, breast cancer, lung cancer, gastrointestinal tract cancers, reproductive system malignancies, and sarcomas. This research impetus is largely driven by the frequent and often elevated expression of stress-inducible NKG2DLs on various solid tumors, underscoring the considerable therapeutic potential of targeting this innate immune pathway. In the following section (**Table 1**), we summarize key preclinical studies of NKG2D CAR-T cells, organized by solid tumor type, and provide a detailed discussion on the current status of preclinical research for tumors originating from different anatomical sites.

**Table 1:** Preclinical data of NKG2D CAR-T in solid tumor with animal data.

Tumor type	CAR construct design	Animal model	Outcome	Reference
GBM	NKG2D extracellular domain (ECD), CD8 hinge and transmembrane (TM) domain, 4-1BB intracellular domain (ICD), and CD3ζ ICD (named NKG2D-BBz CAR)	Five to six-week-old B-NDG mice were subcutaneously (s.c.) injected with stable luciferase transfected U-251MG or U-87MG cells (1×10^6^ cells/each) and received NKG2D-BBz CAR-T cells intravenously (i.v.) (1×10^7^ cells/each) when the mean tumor bioluminescence reached ~5×10^7^ photons/second	NKG2D-BBz CAR-T cells markedly eliminated xenograft tumors, with tumors almost completely diminished at day 21 and no recurrence observed till day 42; the CAR-T cells showed no significant treatment-related toxicity, and their expression did not exert obvious effects on T cell proliferation, apoptosis or genomic stability; they also exhibited potent cytotoxicity against glioblastoma stem cells *in vivo*	(62)
	Human NKG2D extracellular domain (ECD), human CD8α hinge spacer and transmembrane (TM) domain, 4-1BB costimulatory domain, and CD3ζ signaling domain	Six-week-old female B-NDG mice were subcutaneously (s.c.) injected with 2×10^6^ U251 tumor cells; when the tumor volume reached around 100 mm³, the mice were intravenously (i.v.) administered 100 mg/kg sodium valproate (VPA) every two days for 4 times, followed by i.v. injection of 5×10^7^ NKG2D CAR-T cells or untransduced T (UTD) cells	NKG2D CAR-T cells significantly inhibited tumor growth, and VPA pretreatment further enhanced the antitumor effect; no obvious difference in body weight was observed among groups, indicating good safety; the combination therapy remarkably increased CD3^+^ T cell infiltration into tumor sites	(64)
Breast Cancer	Human NKG2D extracellular domain (ECD), CD8α hinge and transmembrane (TM) domain, CD3ζ intracellular domain (ICD) alone (NKG2D-z) or in tandem with 4-1BB ICD (NKG2D-BBz) or CD27 ICD (NKG2D-27z)	Six to ten-week-old female NSG mice were subcutaneously (s.c.) injected with 3×10^6^ luciferase-labeled MDA-MB-231 cells; when the tumor volume reached 200–300 mm³, the mice received intravenous (i.v.) injection of NKG2D CAR-T cells (3×10^7^ cells/each, ~30% CAR+) on day 40 and day 45 after tumor inoculation	NKG2D-BBz and NKG2D-27z CAR-T cells significantly inhibited tumor growth, with tumors significantly smaller than those in the control group and NKG2D-z group (P<0.001); the costimulated CAR-T cells maintained stable and high surface CAR expression *in vivo*, showed enhanced persistence (higher CD4+ and CD8+ T cell counts in peripheral blood at day 20), and migrated to tumor sites; mice in the control group (PBS, UNT) had progressive tumor growth and were euthanized by day 90	(70)
Lung Cancer	NKG2D chimeric antigen receptor, with NKG2D as the extracellular antigen-binding domain targeting NKG2DL	NOG mice were used for murine xenograft assay; target NSCLC cells (PC-9 or NCL-H460 cells) were implanted into mice to establish xenograft models; mice were treated with NKG2D CAR-T cells from diverse human autologous T cell sources (including peripheral blood of healthy donors, H-T cells; peripheral blood of NSCLC patients) or non-transduced T cells (NT-T cells, peripheral blood T lymphocytes of healthy volunteers without NKG2D CAR insertion) as controls	Compared with NT-T cells, NKG2D CAR-T cells (especially H-T cells) significantly diminished xenograft tumors, improved mice survival, increased mice body weight and tumor-infiltrating capacity, and upregulated serum IFN-γ level; the antitumor effect of NKG2D CAR-T cells was NKG2DL-dependent	(71)
	NKG2D extracellular domain (ECD), hinge and transmembrane domain, 4-1BB intracellular domain (ICD), CD3ζ ICD, and co-expressed CX3CR1; EGFRt as tracking and safety ablation marker (control construct: NKG2D-CAR co-expressing IL-15)	Animal model: 6-8-week-old male NOD.Cg-Prkdcscid IL2rgtm1Wjl/SzJ (NSG) mice; subcutaneous injection of 1×10^5^ A549-luc or HepG2-luc cells to establish xenograft models; when tumors were measurable (≈3 weeks later), intravenous injection of 3×10^7^ CAR-T cells (NKG2D-CX3CR1, NKG2D-IL15 or control pCDCAR) twice at 2-month intervals, with intraperitoneal injection of human IL-2 biweekly to maintain T cell viability	Animal experiment outcome: NKG2D-CX3CR1 CAR-T showed stronger tumor growth inhibition and reduced tumor burden than NKG2D-IL15 and control groups; improved mice survival, enhanced CAR-T tumor infiltration, increased serum IFN-γ level, and exhibited less exhausted phenotype (lower PD1 expression) with more naive/central memory T cell subsets	(73)
Lung Cancer and HCC	NKG2D extracellular domain (ECD, recognizes NKG2DLs), DAP10 cytoplasmic domain (mediates signal transduction), CD3ζ intracellular domain (ICD, activating signal), plus CD28 or 4-1BB ICD (costimulatory signal)	6-8-week-old NOD-SCID IL2γ^−^/^−^ (NSI) mice; subcutaneous injection of 5×10^5^ A549GL cells (NSCLC cell-derived xenografts) or 2×2×2 mm³ patient-derived tumor pieces (NSCLC/HCC PDX models); intravenous injection of 5×10^6^ NKG2D-CAR-T cells (or DAP10-enhanced NKG2D-related CAR-T cells) with GFP-T cells as control	Animal experiment outcome: NKG2D-CAR-T cells (especially DAP10-enhanced ones) reduced tumor volume/weight, increased intratumoral T cell infiltration and CAR-T accumulation, continuously secreted IL-2/IFN-γ, and showed no obvious toxicity	(72)
HCC	Human NKG2D extracellular domain (ECD), CD8 hinge and transmembrane domain, 4-1BB costimulatory domain, and CD3ζ intracellular signaling domain	Five to six-week-old B-NDG mice were subcutaneously injected with 1×10^6^ SMMC-7721-luciferase cells (mixed with 30% Matrigel); 7 days later, mice received 1×10^7^ NKG2D-BBz CAR-T cells, nontransduced T cells (NTD), CD19-BBz CAR-T cells or saline via tail vein injection	NKG2D-BBz CAR-T cells significantly suppressed HCC xenograft growth, with 50% of mice tumor-free 19 days post-infusion and 4/6 mice tumor-free at the study end; the cells preferentially infiltrated tumors and showed NKG2D ligand (NKG2DL)-dependent cytotoxicity without obvious off-target toxicity	(57)
Gastric Cancer	NKG2D extracellular portion (amino acids 82–216), CD8α hinge-transmembrane domains, 4-1BB intracellular signaling domain, CD3ζ intracellular signaling domain, followed by eGFP via a F2A ribosomal skipping sequence	Six to eight-week-old female NSG (NOD/scid IL2RG-null) mice were subcutaneously inoculated with 5×10^6^ MKN-28 cells; when tumor volume reached 100–200 mm³, mice (n=6) received 5×10^6^ NKG2D-CAR-T cells, mock-transduced T cells or PBS via caudal vein injection	NKG2D-CAR-T cells significantly inhibited the growth of gastric cancer xenografts, accumulated in tumor sites and suppressed tumor cell proliferation; low-dose cisplatin upregulated NKG2D ligand expression on tumor cells, enhancing the anti-tumor efficacy of NKG2D-CAR-T cells	(76)
	NKG2D extracellular domain (amino acids 82–216), CD8α hinge domain, CD28 transmembrane domain, CD28 and 4-1BB costimulatory domains, CD3ζ intracellular activating domain; PGK promoter-driven IL-15 and CCL19 (linked by 2A sequence) and GFP were introduced	Forty-eight-hour post-fertilization zebrafish were injected with 200 Dil-stained HCG-27 cells into the vitellicle (*in situ* model) or perivitelline space (metastatic model); 24 hours later, the same number of effector cells (untreated, NT, conventional CAR-T, 15×19 CAR-T) were injected at the same site	15×19 CAR-T cells significantly reduced gastric cancer xenograft tumor size in zebrafish, showed obvious expansion *in vivo*, efficiently eliminated *in situ* and metastatic tumor cells, induced tumor cell apoptosis, enhanced T cell proliferation, reduced T cell exhaustion marker (CTLA-4, PD1) expression, and increased central memory T cell proportion	(77)
	Anti-ULBP2 single-chain variable fragment (scFv), human CD8 hinge and transmembrane domains, 4-1BB costimulatory domain, and CD3ζ intracellular signaling domain	Six to eight-week-old female NSG mice were subcutaneously injected with 3×10^6^ MKN-45-ULBP2-T2A-Luc cells (CDX model) or 10 mm³ patient-derived tumor tissues (PDX model); when tumor volume reached ~90 mm³, mice received 6×10^6^ ULBP2 CAR-T cells via intravenous injection, with anti-PD-1 antibody (10 mg/kg, intraperitoneal injection every 5 days) for combination therapy	ULBP2 CAR-T cells alone significantly inhibited tumor growth and prolonged survival in CDX and PDX models; combination with anti-PD-1 further enhanced antitumor efficacy, reduced stromal deposition and CAF activation, promoted CD8^+^ T cell infiltration, and decreased T cell exhaustion markers (PD-1, LAG-3, TIM-3) without obvious toxicity	(58)
	NKG2D extracellular domain (ECD), CD8 hinge and transmembrane (TM) domain, 4-1BB costimulatory domain, CD3ζ intracellular signaling domain (NKG2D-BBz CAR)	Five to six-week-old female NCG mice were subcutaneously injected with 5×10^6^ NCI-N87 or MKN45 cells; when tumor volume reached ~70 mm³, mice received intraperitoneal injection of WAY-262611 (DKK1 inhibitor, 10 mg/kg) every 2 days and intravenous injection of 1×10^7^ NKG2D-CAR-T cells (KD-025) on day 7 post tumor inoculation	Combination therapy of WAY-262611 and NKG2D-CAR-T cells showed superior antitumor efficacy compared to single treatment, significantly inhibiting tumor growth, reducing tumor volume and weight; CAR-T cell infiltration in tumors was enhanced; no obvious treatment-related toxicity or weight loss was observe	(80)
Colorectal Cancer	CD8α signal sequence, human NKG2D extracellular domain (amino acids 82–216), CD8α hinge region, CD28 transmembrane and intracellular domain, 4-1BB intracellular signaling domain, CD3ζ intracellular signaling domain; encoded by non-viral minicircle DNA vector with GFP sequence	Four to six-week-old male NOD/SCID mice were subcutaneously injected with 1×10^6^ HCT-116-Luc cells; when tumor volume reached 150–250 mm³, mice received 1×10^7^ NKG2D CAR-T cells, untransduced T cells or PBS via tail vein injection on day 0 and 7	NKG2D CAR-T cells significantly suppressed tumor growth, reduced tumor volume and prolonged overall survival of mice; human NKG2D-positive lymphocytes infiltrated tumor tissues; no severe pathological changes were found in vital organs	(78)
Pancreatic Cancer	Second-generation NKG2D-CAR: CD8α signal peptide, NKG2D extracellular domain, CD8α hinge and transmembrane domain, 4-1BB intracellular domain, CD3ζ intracellular domain; co-expressed with shRNA2-4.1R (KD2) for 4.1R knockdown	Eight-week-old female NSG mice were subcutaneously injected with 6×10^6^ PANC28-luc cells; when tumors formed (around day 10), mice received 1×10^7^ KD2-NKG2D-CAR-T cells, NKG2D-CAR-T cells, NC-NKG2D-CAR-T cells, Mock T cells or PBS via tail vein injection	KD2-NKG2D-CAR-T cells significantly suppressed tumor growth, reduced tumor volume, and prolonged mouse survival compared to other groups; peripheral blood CD3^+^ T cell counts and effector memory T cell ratio were higher; no obvious severe toxicity was observed; the enhanced antitumor activity was mediated by ERK signaling pathway	(85)
	① NKG2D-CAR (second-generation): Murine NKG2D extracellular and transmembrane domains, 4-1BB intracellular domain, CD3ζ intracellular domain; ② Bicistronic CAR: anti-MUC1 CAR linked via P2A element to NKG2D-CAR	Six-week-old C57 mice were used to establish orthotopic PDAC models via tumor slice transplantation (TST) (Panc02 tumor slices implanted into pancreatic subcapsular space) and heterotopic models via subcutaneous inoculation; NCG mice were also used for related experiments	NKG2D-CAR-T cells specifically eliminated myeloid-derived suppressor cells (MDSCs) expressing NKG2D ligand Rae1; combination of NKG2D-CAR-T and aMUC1-CAR-T significantly prolonged orthotopic model mice survival (40% survived >100 days); bicistronic CAR-T also exerted potent antitumor efficacy, while single aMUC1-CAR-T or PD1/CTLA4 blockade showed poor response to orthotopic tumors	(86)
	① NKG2D-CAR (second-generation): Human NKG2D extracellular domain (amino acids 82–216), CD8α hinge and transmembrane domain, 4-1BB intracellular domain, CD3ζ intracellular domain; ② 4/15NKG2D-CAR: NKG2D-CAR co-expressing IL-4R extracellular domain and IL-15R transmembrane/intracellular domain via T2A sequence (inverted cytokine receptor, converting IL-4 inhibitory signals to IL-15 activation signals)	Eight-week-old female NSG mice were subcutaneously injected with 5×10^6^ Panc-1-Luc cells; when tumor volume reached 150–200 mm³, mice received intravenous injection of 4/15NKG2D-CAR-T cells, NKG2D-CAR-T cells, Mock T cells or PBS	4/15NKG2D-CAR-T cells significantly suppressed tumor growth, reduced tumor volume and prolonged mouse survival compared to NKG2D-CAR-T cells; enhanced CD3^+^ T cell infiltration in tumors and increased proportion of central memory T cells (CD45RO^+^CD62L^+^); no obvious severe toxicity was observed	(87)
	Second-generation NKG2D-CAR: Human NKG2D extracellular domain, CD8α hinge and transmembrane domain, 4-1BB intracellular domain, CD3ζ intracellular domain; co-expresses IL-15/IL-15Ra complex (IL15C, IL-15 linked to IL-15Ra sushi domain) via T2A sequence	Eight-week-old female NSG mice were subcutaneously injected with 5×10^6^ PANC1-Luc cells; when tumor volume reached 100–300 mm³, mice received intravenous injection of 1×10^7^ IL15C-NKG2D-CAR-T cells, NKG2D-CAR-T cells, Mock T cells or PBS	IL15C-NKG2D-CAR-T cells significantly suppressed tumor growth, reduced tumor volume and prolonged mouse survival compared to NKG2D-CAR-T cells; enhanced CD3^+^ T cell and CD8^+^ T cell infiltration in tumors, reduced PD-1/TIM3 expression (less exhaustion), increased central memory T cell (CD45RA^−^CCR7^+^) proportion; the enhanced antitumor activity was mediated by JAK3/STAT5 signaling pathway, with no obvious severe toxicity	(88)
Ovarian Cancer	Chimeric NKG2D receptor (chNKG2D):Full-length human/murine NKG2D sequence fused to the cytoplasmic domain of CD3ζ (first-generation CAR, no costimulatory domain)	Six to eight-week-old female C57BL/6mice were intraperitoneally injected with 1×10^6^ ID8-GFP murine ovarian cancer cells; 7 days later, mice received intraperitoneal injection of 5×10^6^ chNKG2D-CAR-T cells, wtNKG2D-T cells or PBS	chNKG2D-CAR-T cells significantly reduced ovarian cancer burden in mice, decreasing the percentage of GFP^+^ tumor cells in peritoneal wash and the number of solid peritoneal tumors; no obvious treatment-related toxicity was observed; the cells secreted proinflammatory cytokines (IFN-γ, GM-CSF, TNF-α) and chemokines (CCL3, CCL5) when co-cultured with tumor cells	(55)
	Chimeric NKG2D receptor (chNKG2D): NKG2D extracellular domain fused to CD3ζ cytoplasmic signaling domain (first-generation CAR, no costimulatory domain)	Seven to ten-week-old female C57BL/6 mice (and CXCR3^−^/^−^, IFNγ^−^/^−^, MHC Class II^−^/^−^, CD8^−^/^−^ knockout mice) were intraperitoneally injected with 2×10^6^ ID8-derived cells; 5 weeks later, mice received intraperitoneal injection of 5×10^6^ chNKG2D-CAR-T cells, wtNKG2D-T cells or PBS	chNKG2D-CAR-T cells significantly reduced solid tumor number and free tumor cells in peritoneal cavity, prolonged mouse survival; optimal antitumor effect relied on host CD4^+^ and CD8^+^ T cells; secreted IFNγ/GM-CSF to induce CXCL9/CXCL10 production, recruiting endogenous T cells via CXCR3; host CD4^+^ T cells were critical for tumor-specific memory response, while CD8^+^ T cells were necessary for optimal tumor elimination; no obvious treatment-related toxicity was observed	(91)
Cervical Cancer	Second-generation NKG2D-CAR: Human NKG2D extracellular domain, CD8α hinge and transmembrane domain, 4-1BB costimulatory domain, CD3ζ intracellular signaling domain (lentiviral vector expression, transduction efficiency ~85.9%)	Six-week-old NOG mice were subcutaneously injected with 4×10^6^ SiHa cells; 14 days later, mice received intravenous injection of 2×10^6^ NKG2D-CAR-T cells, non-transduced T cells (NT-T) or PBS	NKG2D-CAR-T cells specifically killed NKG2DL-positive cervical cancer cells *in vitro* (dose-dependent cytotoxicity), secreted higher levels of IFN-γ, and upregulated CD107a expression; *in vivo*, they significantly suppressed tumor growth, prolonged mouse survival, and infiltrated tumor tissues robustly; no obvious off-target toxicity was observed (no CAR-T retention in major organs, no significant body weight changes)	(93)
Prostate Cancer	Second-generation NKG2D-CAR: Human NKG2D extracellular domain (amino acids 82–216), CD8α hinge and transmembrane domain, 4-1BB costimulatory domain, CD3ζ intracellular signaling domain; NKG2DIL7-CAR additionally co-expresses IL-7 via T2A sequence (lentiviral vector expression)	Six to eight-week-old female NSG mice were subcutaneously injected with 2×10^6^ PC-3 cells; when tumor volume reached 150–200 mm³, mice received intravenous injection of 1×10^7^ NKG2DIL7-CAR-T cells, NKG2D-CAR-T cells, non-transduced T cells (NT-T) or PBS	NKG2DIL7-CAR-T cells showed stronger *in vitro* cytotoxicity against prostate cancer cells (E:T ratio-dependent) than NKG2D-CAR-T cells; enhanced CD8^+^ T cell proliferation, upregulated Bcl-2 and Glut1 expression, reduced apoptosis and exhaustion (lower PD-1/Tim-3 expression); *in vivo*, significantly suppressed tumor growth, reduced tumor volume/weight, prolonged mouse survival, increased CD8^+^ T cell infiltration and central memory T cell (CD45RA^+^CCR7^+^) proportion in tumors; no obvious severe toxicity was observed	(53)
	Second-generation NKG2D-CAR: Human NKG2D extracellular domain, CD8α hinge and transmembrane domain, 4-1BB costimulatory domain, CD3ζ intracellular signaling domain; integrated with HDAC11-targeting shRNA (shD sequence, optimal interference efficiency) to generate shD-NKG2D-CAR (lentiviral vector expression)	Eight-week-old female NSG mice were subcutaneously injected with 5×10^6^ PC-3-luciferase cells; when tumor volume reached 100–300 mm³, mice received intravenous injection of 1×10^7^ shD-NKG2D-CAR-T cells, NKG2D-CAR-T cells, Mock-T cells or PBS	shD-NKG2D-CAR-T cells showed enhanced *in vitro* cytotoxicity against prostate cancer cells (E:T ratio-dependent), elevated CD69/CD107a expression (activation/degranulation), increased IFN-γ/GzmB secretion, enhanced proliferation, reduced PD-1/TIM3 expression (less exhaustion), and increased central memory T cell (Tcm) proportion via upregulating Eomes; *in vivo*, significantly suppressed tumor growth, reduced tumor volume/bioluminescence intensity, prolonged mouse survival, enhanced CD3^+^ T cell tumor infiltration, and improved circulating CAR-T cell functional persistence (lower exhaustion, higher CD8^+^ T cell and Tcm ratios); no obvious severe toxicity was observed	(94)
Osteosarcoma	Second-generation NKG2D-CAR: Human NKG2D extracellular domain, CD8α hinge and transmembrane domain, 4-1BB costimulatory domain, CD3ζ intracellular signaling domain; expressed in CD45RA^−^ memory T cells (minimal alloreactivity) via lentiviral vector	Ten to twelve-week-old NOD/scid IL2Rγ^−^/^−^ (NSG) mice were intra-tibially injected with 5×10^5^ 531MII YFP-luc cells; mice received intra-tibial or intravenous injection of 5×10^6^ NKG2D-CAR^+^ CD45RA^−^ T cells, untransduced CD45RA^−^ T cells or no treatment, with intraperitoneal injection of human IL-2 (100 IU/mouse) every other day for 3 weeks	NKG2D-CAR^+^ CD45RA^−^ T cells showed significantly enhanced *in vitro* cytotoxicity against osteosarcoma cells (E:T ratio-dependent) compared to untransduced T cells; *in vivo*, they reduced tumor burden (lower bioluminescent signal), prolonged mouse survival (up to 120 days), and induced protective immunity against tumor rechallenge; no obvious toxicity (no GVHD, CRS, or liver damage), no chromosomal aberrations, and minimal cytotoxicity against healthy adult tissues (except fetal intestine cell line Hs1.Int and immortalized lung cell line NuLi-1)	(101)

### 4.1 Glioblastoma and neuroblastoma

NKG2D CAR-T cells have shown considerable therapeutic potential in preclinical models for glioblastoma (GBM), a tumor characterized by high heterogeneity and a profoundly immunosuppressive microenvironment (59). NKG2DLs are broadly expressed in GBM, with enrichment in CD133^+^ glioma stem-like cells (GSCs), underscoring their relevance as immunotherapeutic targets (18, 60). Radiation-induced DNA damage has been shown to increase NKG2DL expression, thereby enhancing immune recognition and cytotoxicity (61). Importantly, NKG2D CAR-T cells effectively eliminated both bulk GBM cells and CD133^+^ GSCs, markedly reducing recurrence and demonstrating the potential to overcome tumor heterogeneity and resistance (62). To address the fratricide issue during NKG2D CAR-T cell amplification, a next-generation construct (CYAD-02) was developed, co-expressing shRNAs against MICA/B to suppress endogenous ligand expression, which improved persistence and cytotoxic efficacy *in vivo (*63). Epigenetic modulation has also been explored as a sensitization strategy. Using the histone deacetylase(HDAC) inhibitor sodium valproate (VPA), a clinically approved antiepileptic agent, significantly increased surface NKG2DLs expression on GBM cells at a sublethal concentration. VPA treatment enhanced the susceptibility of GBM cells to NKG2D CAR-T mediated cytotoxicity in both 2D monolayer and 3D tumor spheroid models *in vitro*. Moreover, VPA-treated GBM cells stimulated CAR-T cells to produce higher levels of inflammatory cytokines (IL-2, IFN-γ, and IL-6). *In vivo*, this combination therapy resulted in robust tumor growth inhibition in B-NDG xenograft mouse models (64).

Compared to other solid tumors, progress in CAR-T cell therapy for neuroblastoma has been relatively limited (65, 66). Although neuroblastoma cells commonly express NKG2DLs (including MICA/B and ULBP1-3), their levels are frequently suppressed by oncogenic pathways such as MYCN amplification, which suppresses NKG2DL transcription, thereby reducing tumor immunogenicity and promoting resistance to NK and T cell-mediated cytotoxicity (67). To date, no systematic preclinical studies on NKG2D CAR-T cell therapy for neuroblastoma have been reported. However, emerging evidence indicates that CAR-NK cells can effectively eradicate tumor-associated MDSCs, thereby enhancing the antitumor activity of CAR-T cells. These findings exploration of novel combination immunotherapy strategies, such as dual CAR constructs or staged treatment regimens, to provide innovative therapeutic approaches for targeting neuroblastoma (68).

### 4.2 Breast cancer

Triple-negative breast cancer (TNBC) cells broadly upregulate NKG2DLs including MICA, MICB, and ULBP1-4, in response to cellular stress, offering a promising target for the development of novel immunotherapies against this aggressive subtype (18, 69). Preclinical studies have evaluated multiple CAR configurations: a first-generation NKG2D-z (containing only the CD3ζ signaling domain), and second-generation NKG2D-27z and NKG2D-BBz (incorporating CD27 or 4-1BB co-stimulatory domains, respectively) (70). All these CARs utilized the extracellular domain of human NKG2D (amino acids 82-216) as the antigen recognition element, fused via a CD8α hinge and transmembrane region to the respective intracellular signaling domains. Although NKG2D-z CAR-T cells showed self-expansion under IL-2 stimulation, they exhibited limited cytotoxic activity, whereas NKG2D-27z and NKG2D-BBz CAR-T cells demonstrated significantly enhanced antitumor efficacy, with improved cytotoxicity, cytokine secretion, and tumor suppression in both *in vitro* and *in vivo* models. A recent analysis using an NKG2D-Fc fusion protein system profiled MICA, MICB, and ULBP1–4 across eight TNBC cell lines, identifying MICA and ULBP2 as the predominant ligands (69). Although this study focused on bispecific fusion proteins rather than NKG2D CAR-T cells, the findings further support the therapeutic relevance and stable expression of NKG2DLs as promising targets in TNBC immunotherapy.

### 4.3 Lung cancer

Preclinical studies have highlighted the therapeutic promise of NKG2D CAR-T cells in lung cancer. In non-small cell lung cancer (NSCLC) models, they displayed potent cytotoxicity *in vitro* and induced robust tumor suppression, prolonged survival, and enhanced T-cell infiltration *in vivo*, effects closely linked to elevated NKG2DL expression (71). These effects were highly dependent on elevated NKG2DL expression in NSCLC cells, indicating that NKG2D CAR-T cells represent a promising candidate therapeutic approach with substantial targeting potential for solid tumors. To further optimize functionality, a modified construct (M28z10) incorporating the DAP10 cytoplasmic domain into a second-generation mesothelin-targeting CAR enhanced cytotoxicity, cytokine secretion (IL-2, IFN-γ, granzyme B), and durable anti-tumor responses in both A549GL xenograft models and patient-derived xenograft (PDX) models (72). Flow cytometry analysis revealed that M28z10-CAR-T cells exhibit enhanced activation (CD25^+^/CD69^+^) and degranulation capacity (CD107a^+^), with minimal impact on PD-1 expression levels, suggesting their ability to maintain functional activity within the TME. Further engineering efforts have focus on enhancing CAR-T cell tumor infiltration capacity. Researchers revealed that downregulation of the CX3CL1-CX3CR1 axis in NSCLC restricts the infiltration of cytotoxic lymphocytes into tumor sites (73). Therefore, upregulating CX3CR1 expression in NKG2D CAR-T cells theoretically enhances their tumor infiltration capacity. Although this review focuses on CAR-T cells, recent CAR-NK studies in lung cancer offer valuable mechanistic insights. For instance, engineering CAR-NK cells to simultaneously target PD-L1 and MICA/B, or to constitutively express IL-21 to enhance the cytotoxicity and proliferation, highlights cytokine-mediated pathway activation as a promising strategy to enhance NKG2D signaling, offering valuable insights for next-generation CAR-T cell design (74, 75).

### 4.4 Gastric and colorectal cancer

Preclinical development of NKG2D CAR-T cell therapy for gastric and colorectal cancers has advanced rapidly, with efforts converging on three main fronts: target selection, structural optimization, and combination strategies.

In gastric cancer, second-generation NKG2D CAR-T cells have been shown to target broadly expression MICA/B and ULBP1–3 across multiple gastric cancer cell lines, achieving marked tumor suppression in xenograft models (76). Moreover, cisplatin co-treatment upregulates NKG2DLs expression on gastric cancer cells, enhancing the cytotoxic activity of NKG2D CAR-T cells. Incorporation of IL-15 and CCL19 into the CAR construct further enhanced expansion, central memory differentiation, cytokine secretion, bystander T-cell recruitment, and resistance to exhaustion, enabling potent and sustained tumor clearance at both primary and metastatic sites with minimal off-tumor toxicity (77).

Colorectal cancer has advanced with comparable momentum. Third-generation NKG2D CAR-T cells demonstrated durable antitumor activity *in vitro* and *in vivo* without evident histopathological toxicity (78). Second-generation architecture (NKG2D ectodomain-CD8α hinge-CD28 transmembrane-4-1BB-CD3ζ) achieved marked tumor regression and extended survival in xenograft models, with histological confirmation of robust intratumoral infiltration (79). Combination therapy with the DKK1 inhibition (WAY-262611) remodeled the immunosuppressive TME, dose-dependently upregulated NKG2DL expression, enhanced cytotoxicity by up to sixfold, and increased IFN-γ/TNF-α secretion. *In vivo*, this strategy reduced tumor burden by more than 50% compared with monotherapy and improved CAR-T cell penetration into tumor parenchyma (80). To mitigate systemic toxicities and risks associated with persistent CAR expression, a transient mRNA-based NKG2D-CAR platform was developed to enable controlled expression and activity. In a colorectal cancer peritoneal metastasis model, intraperitoneal delivery of these short-lived CAR-T cells achieved significant tumor control without chronic toxicity (81). Moreover, the immunomodulatory drug lenalidomide significantly enhanced NKG2D CAR-T cell proliferation, cytotoxicity, and Th1 polarization by promoting degradation of the transcriptional repressors Ikaros and Aiolos, thereby activating AP-1 and ERK signaling. This combinatorial approach offers a potential means to overcome tumor microenvironment-mediated suppression, although clinical translation will require careful management of CRS risk and further dissection of NKG2D downstream signaling to optimize the therapeutic window (79).

### 4.5 Pancreatic cancer

Pancreatic cancer is a highly immunosuppressive solid malignancy with poor early detection rates. NKG2D CAR-T cell therapy is undergoing continuous innovation, with engineering designs primarily focused on enhancing functional persistence and tumor infiltration capacity within the desmoplastic TME (82–84).

A second-generation CAR incorporating shRNA-mediated silencing of the 4.1R gene (EPB41) reduced PD-1/TIM-3 expression, promoted proliferation, and increased granzyme B/IFN-γ secretion. These cells exhibited enhanced cytotoxicity against PANC-28 and CAPAN-2 *in vitro*, suppressed tumor growth *in vivo*, and prolonged survival via ERK pathway activation (85). To address the core immunosuppressive features of pancreatic ductal adenocarcinoma (PDAC), an orthotopic xenograft model established by tissue slice transplantation demonstrated a dual-track strategy. NKG2D CAR-T cells selectively depleted ULBP1^+^ MDSCs, alleviating immunosuppressive barriers, while bifunctional αMUC1/NKG2D CAR-T cells induced durable tumor control, including 50% complete remission in advanced disease (86). Other approaches have tackled cytokine-mediated immunosuppression. A novel inverted cytokine receptor (ICR) strategy has been developed to counter the profound immunosuppressive effects of IL-4-abundantly expressed in the pancreatic TME thereby preventing IL-4 induced CAR-T cell exhaustion. This approach preserves target specificity while enhancing cytotoxicity, upregulating activation (CD69^+^) and degranulation (CD107a^+^) markers, increasing effector cytokine secretion, strengthening anti-apoptotic capacity, and sustaining memory phenotypes. *In vivo*, ICR-engineered CAR-T cells achieved effective control of tumor progression (87). Cytokine engineering has also shown promise: a chimeric IL-15 complex (IL-15C), generated by fusing IL-15 to the sushi domain of IL-15Rα, enhanced receptor engagement, amplified effector function, and reprogrammed T-cell differentiation (88).

### 4.6 Ovarian and cervical cancers

Ovarian and cervical cancers are among the leading causes of cancer-related mortality in gynecologic malignancies and are characterized by profoundly immunosuppressive TME (89, 90). MICA/B and ULBP2 expression was detected in 97.6% and 82.9% of ovarian cancer cells respectively, but absent in normal ovarian epithelium. In cervical cancer, MICA/B, ULBP1, and RAET1E (ULBP4) are exceeded at significantly higher levels than in low-grade cervical intraepithelial neoplasia (CIN) or normal cervical tissues. These findings collectively establish NKG2DLs as actionable targets for immunotherapy in ovarian and cervical cancers (19–21).

Preclinical studies in ovarian cancer demonstrated that NKG2D ectodomain and CD3ζ signaling module (chNKG2D) induced complete remission in murine ovarian cancer and generated durable immune memory. Critically, researchers observed immunogenic remodeling of the TME, characterized by a phenotypic shift of tumor-associated myeloid cells from immunosuppressive to immune-activating states, accompanied by elevated expression of pro-inflammatory cytokines such as IFN-γ and GM-CSF (55). Acting as “immunological primers” NKG2D CAR-T cells activated antigen-presenting cells and recruited host T cells via CXCR3-dependent chemotaxis, generating antigen-specific memory responses and suggesting a synergistic paradigm that may obviate lymphodepletion (91). At the cellular level, ovarian cancer lines and primary tumors consistently express MICA/B and ULBPs, rendering them susceptible to selective lysis by NKG2D CAR-T cells while sparing ligand-negative controls (92). Moreover, ligand-low tumors can be sensitized with HDAC inhibitors such as valproic acid(VPA), which upregulate NKG2DLs and enhance CAR-T recognition without impairing T-cell viability (18).

In cervical cancer, second-generation NKG2D-BBζ-CAR effectively recognized and lysed NKG2DL cervical cancer cells while sparing ligand-negative C-33A cells. Increased CD107a expression confirmed enhanced activation and degranulation, and *in vivo* studies showed moderate but significant tumor suppression (93). Clinically, tissue microarray analysis of 200 cervical cancer samples revealed that high MICA/B and ULBP1 expression correlated with prolonged progression-free and overall survival, whereas elevated RAET1E (ULBP4) and RAET1G (ULBP5) predicted poorer outcomes (21).

### 4.7 Prostate cancer

In prostate cancer, engineering strategies for NKG2D CAR-T cells have focused on enhancing persistence, functional fitness, and resistance to tumor-induced immunosuppression. Corporation of a human IL-7 gene into second-generation NKG2D CAR-T cells improved survival and cytotoxicity against NKG2DL^+^ prostate cancer cell lines, induced robust apoptosis in co-culture assays, and suppressed tumor growth in NSG xenografts, correlating with elevated Bcl-2 expression, reduced PD-1 levels, and an enriched central memory phenotype (53). Epigenetic enhancement via HDAC11 knockdown further optimized antitumor activity, with shD-NKG2D CAR-T cells showing superior cytotoxicity against PC-3 and DU-145 cells, increased Eomes expression, augmented proliferation, reduced PD-1/TIM-3 expression, and sustained central memory differentiation-indicative of improved functional persistence and immunological memory (94). Moreover, suppression of the NKG2D pathway by the immunosuppressive TME is a key mechanism of immune evasion in prostate cancer (95–97). Prostate cancer cells secrete exosomes bearing MICA/B and ULBP2, which bind to and downregulate NKG2D on NK and CD8^+^ T cells, thereby attenuating cytotoxic responses. Blocking exosomal NKG2DLs partially restored NKG2D expression, and reduced NKG2D levels were confirmed in circulating lymphocytes from castration-resistant prostate cancer patients. This exosome-mediated immune evasion may underlie limited clinical responses to NKG2D CAR-T cells, highlighting the need for strategies to counteract ligand shedding and restore NKG2D signaling (98).

### 4.8 Other solid tumors

Studies have demonstrated that NKG2DLs are stress-inducibly overexpressed in hepatocellular carcinoma (HCC) cells, providing attractive therapeutic targets for NKG2D CAR-T cell-based treatment of hepatic solid tumors (18). A systematic preclinical evaluation in 2019 demonstrated that second-generation NKG2D CAR-T cells selectively eliminated high-NKG2DL-expressing HCC cells *in vitro*, with target-dependent killing confirmed through ligand overexpression and knockout models (57). Notably, NKG2D’s broad ligand recognition may mitigate immune escape caused by antigenic heterogeneity, while retaining autonomous antitumor activity in lymphopenic settings lacking adaptive immunity. Building on this, dual-specific GC3328z-NKBB CAR-T cells combining a glypican-3 (GPC3) CAR with an NKG2D (NKBB) receptor enabled simultaneous targeting of GPC3 and NKG2DLs. The NKBB module enhanced migration, intratumoral infiltration, and central memory T cell expansion while reducing exhaustion, yielding durable and potent anti-HCC responses (99).

In osteosarcoma, NKG2DLs (MICA/B and ULBPs) are widely expressed but display substantial spatial heterogeneity within a profoundly immunosuppressive TME, resulting in limited infiltration, rapid functional exhaustion, and potential off-tumor cytotoxicity. Epigenetic priming with the HDAC inhibitor (VPA) increased surface MICA/B expression and sensitized tumor cells to NK cell-mediated cytotoxicity (100). Second-generation CAR-T cells demonstrated robust *in vitro* cytotoxicity against MG-63 and U-2 OS cells and delayed tumor progression in orthotopic intraosseous xenografts (101). More recently, a next-generation C5/IL7-CAR co-expressing CXCR5 and IL-7 enhanced activation, degranulation, and cytokine production, while downregulating exhaustion markers (PD-1, TIM-3, TIGIT) and upregulating Bcl-2. This configuration promoted stem-like memory differentiation, improved intratumoral infiltration, and achieved superior survival outcomes in osteosarcoma models compared with conventional CAR-T cells (102).

## 5 Clinical response heterogeneity: application status and site-specific challenges across anatomical locations

Current clinical exploration of NKG2D CAR-T cell therapy for solid tumors is progressing steadily, with several programs advancing into early-phase trials. Based on the information from clinicaltrial.gov, We have summarized the clinical data of NKG2D CAR-T **Table 2**. However, efficacy varies markedly by anatomical site, reflecting tumor-specific biological barriers and underscoring the importance of tailored engineering strategies. Clinical trial registries list NKG2D CAR-T cells and their derivative constructs under evaluation for multiple solid tumors, including colorectal cancer, ovarian cancer, gastric cancer, HCC, breast cancer, prostate cancer, and GBM multiforme, predominantly in patients with advanced or metastatic refractory disease (103). In colorectal cancer patients with peritoneal metastases, intraperitoneal administration of NK cells transiently expressing NKG2D-CAR via mRNA electroporation rapidly induced ascites and shrank tumor burden, underscoring the therapeutic potential of localized administration for spatially constrained solid tumors (e.g., peritoneal carcinomatosis) and establishing a novel therapeutic paradigm for CAR-based therapies (81). In gastric and HCC, CAR constructs incorporating a CD8α hinge-4-1BB-CD3ζ architecture improved T cell expansion and *in vivo* antitumor activity (57, 80). In prostate cancer models, IL-7-expressing NKG2D CAR-T cells sustain T cell viability with reduced exhaustion phenotypes, demonstrating superior expansion kinetics and persistence within immunosuppressive TME (53). Notably, in glioblastoma, intravenously administered CAR-T cells successfully traversed the blood-brain barrier (BBB) and accumulated at tumor sites, mediating sustained secretion of IFN-γ and granzyme B. This demonstrates the paradigm-shifting potential of NKG2D CAR-T cell therapy for central nervous system (CNS) malignancies. In 2022, clinical-grade manufacturing of CYAD-101 was achieved, a first-in-class, non-gene-edited allogeneic CAR-T cell therapy based on the NKG2D receptor (104).

**Table 2:** Clinical data of NKG2D CAR-T in solid tumor.

Trial number	Drug Name	Sponsor name	CAR construct	Indication	Outcome	Reference
NCT03310008	CYAD-01	Celyad Oncology	Autologous CAR T-cell product consisting of full-length human NKG2D receptor fused with the human CD3ζ ICD	Colorectal cancer with potentially resectable liver metastases	Unknown status	https://clinicaltrials.gov/study/NCT03310008
NCT03370198	CYAD-01	Celyad Oncology	Autologous CAR T-cell product consisting of full-length human NKG2D receptor fused with the human CD3ζ ICD	Unresectable liver metastases from colorectal cancer (LINK)	Terminated	https://clinicaltrials.gov/study/NCT03370198
NCT03692429	CYAD-101	Celyad Oncology	Allogeneic NKG2D CAR-T	Unresectable metastatic colorectal cancer administered after standard chemotherapy	Recruiting	https://clinicaltrials.gov/study/NCT03692429
NCT04107142		CytoMed Therapeutics	Allogeneic NKG2DL-targeting CAR-γΔT	R/R solid tumor	Unknown status	https://clinicaltrials.gov/study/NCT04107142
NCT04270461		Jiujiang University Affiliated Hospital	NKG2D CAR-T with CD8 hinge region and TM region, 4-1BB ICD and CD3ζ ICD	r/r NKG2DL+ Solid Tumors	Withdrawn	https://clinicaltrials.gov/study/NCT04270461
NCT04550663		The Affiliated Nanjing Drum Tower Hospital of Nanjing University Medical School	NKG2D CAR-T	r/r NKG2DL+ Solid Tumors	Unknown status	https://clinicaltrials.gov/study/NCT04550663
NCT04717999		UWELL Biopharma	NKG2D CAR-T	Recurrent glioblastoma	Unknown status	https://clinicaltrials.gov/study/NCT4717999
NCT04991948	CYAD-101	Celyad Oncology	Allogeneic NKG2D CAR-T	Metastatic colorectal cancer	Recruiting	https://clinicaltrials.gov/study/NCT04991948
NCT05131763		Fudan University	NKG2D CAR-T with CD8 hinge region and TM region, 4-1BB ICD and CD3ζ ICD	r/r NKG2DL+ Solid Tumors	Unknown status	https://clinicaltrials.gov/study/NCT05131763
NCT05248048		TheThird Affiliated Hospital of Guangzhou Medical University	NKG2D CAR-T	Previously Treated Liver Metastatic Colorectal Cancer	Unknown status	https://clinicaltrials.gov/study/NCT05248048
NCT05382377		Jianming Xu	NKG2D CAR-T	Advanced NKG2DL+ solid tumors	Recruiting	https://clinicaltrials.gov/study/NCT05382377
NCT05583201		Jianming Xu	NKG2D/CLDN1 8.2-based CAR-T	Advanced NKG2DL+/CLDN18.2+ solid tumors	Recruiting	https://clinicaltrials.gov/study/NCT05583201
NCT05837299		Changhai Hospital	NKG2D CAR-T	CLDN18.2 positive advanced solid tumors	Recruiting	https://clinicaltrials.gov/study/NCT05837299
NCT05976906		Zhejiang University	Dual-target NKG2D-NKp44 CAR-T	Advanced solid tumors	Unknown status	https://clinicaltrials.gov/study/NCT05976906
NCT06087341		Antonio Pérez Martínez, Institutode Investigación Hospital Universitario La Paz (Responsible Party)	Memory T cells expressing NKG2D-CAR	Advanced sarcoma	Recruiting	https://clinicaltrials.gov/study/NCT06087341
NCT06134960		Peking University	NKG2D/CLDN1 8.2-based CAR-T	Advanced NKG2DL+/CLDN18.2+ solid tumors	Not yetrecruiting	https://clinicaltrials.gov/study/NCT06134960
NCT06193902		Leucid Bio	Lateral NKG2D CAR-T with complementary signaling domains integrated in parallel across the cell membrane	NKG2DL-expressing solid tumors	Recruiting	https://clinicaltrials.gov/study/NCT06193902
NCT06509490		Cancer Institute and Hospital, Chinese Academy of Medical Sciences, Beijing, China	NKG2D CAR-T	Advanced NKG2DL+ solid tumors	Recruiting	https://clinicaltrials.gov/study/NCT06509490

Despite these advances, three key challenges have emerged. First, the broad recognition spectrum of NKG2D, while providing pan-tumor coverage against antigenic heterogeneity, poses potential on-target/off-tumor toxicity risks against stressed normal cells in tissues like the intestine, alveoli, and liver particularly when activation thresholds are not strictly regulated. Second, the expression intensity and cleavage status of NKG2DLs exhibit profound spatial heterogeneity across tumor sites, with elevated soluble MICA/B (sMICA/sMICB) levels in some solid tumors impairing CAR-T cell activity and inducing exhaustion, necessitating combinatorial approaches like ADAM protease inhibitors to restore membrane-bound ligand integrity. Third, the immunosuppressive features of the TME vary drastically across organs, where factors such as TGF-β, IDO, and Tregs are highly enriched in liver and prostate cancers, significantly hindering CAR-T cell expansion and infiltration demanding co-expression of potentiators (IL-7, IL-15, CXCR3) or PD-1 blockade to overcome suppression (8, 105).

The therapeutic profile is further shaped by CAR structural parameters, such as co-stimulatory domain selection (e.g., CD27 versus 4-1BB) and cytokine armoring (e.g., BBζ versus BBζ-IL-15). No standardized design universally optimizes efficacy across all solid tumor contexts. Therefore, while NKG2D CAR-T cells have shown encouraging safety and preliminary efficacy across multiple tumor types, optimal clinical benefit will require site-specific, precision-engineered strategies moving beyond broad-spectrum recognition toward tailored combinations that account for ligand distribution, TME characteristics, and delivery route.

## 6 Future directions and combination therapy strategies

NKG2D CAR-T cell therapy has moved well beyond simplistic receptor designs focused solely on targeted cytotoxicity. Driven by recent breakthroughs and innovative conceptual advances, it is now emerging as one of the most versatile and adaptable platforms for solid tumor immunotherapy (106, 107). Building on existing mechanistic insights and preclinical evidence, future strategies should focus on several critical dimensions.

### 6.1 CAR-T cells targeting single NKG2DLs

Given the ubiquitous expression of NKG2DLs-including MICA, MICB, and ULBP1–6 on malignant cells, NKG2D CAR-T cell therapy has emerged as a compelling strategy for targeting solid tumors (9–14). Unlike conventional CAR-T cells directed single antigens, NKG2D CAR-T cells recognize multiple ligands, conferring a broader therapeutic spectrum. However, the heterogeneous expression of NKG2DLs across tumor types and their diverse presence in normal tissues pose formidable challenges (105).

One promising approach is the development of CAR-T cells engineered to selectively target individual NKG2DLs with distinct functional profiles, such as ULBP2, thereby increasing tumor specificity while reducing off-tumor toxicity. ULBP2 is overexpressed in gastric cancer and drives tumor progression by activating TGF-β-mediated CAF activation and collagen deposition, fostering an immunosuppressive TME. ULBP2 CAR-T cells demonstrated dual activity by eliminating malignant cells and remodeling the TME, thereby enhancing immune infiltration and antitumor efficacy (58).

The pan-ligand approach (e.g., NKG2D-CD3ζ) offers the advantage of broadly targeting multiple NKG2DLs, thereby effectively addressing tumor heterogeneity and making it suitable for tumors with widespread or undefined ligand expression, such as refractory solid tumors. However, this broad reactivity increases the risk of on-target/off-tumor toxicity due to NKG2DL expression on stressed normal tissues, potentially resulting in adverse effects such as hepatotoxicity and myelosuppression. In contrast, the single-ligand strategy (e.g., targeting ULBP2) enhance tumor specificity and reduce off-target risks by focusing on an individual ligand. ULBP2 is highly expressed in gastric and ovarian cancers but minimally or rarely expressed in gliomas (18–20). Nevertheless, the clinical applicability of this approach may be limited by reduced adaptability to tumor heterogeneity, as low or heterogeneous expression of the target ligand can facilitate immune escape.

In clinical practice, pan-ligand strategies are better suited for tumors with broad NKG2DL expression and without clearly actionable targets, whereas the single-ligand approach requires precise patient stratification based on ligand expression profiles to optimize both efficacy and safety. Future efforts should focus on the development of “precision single-ligand” CAR-T cells capable of targeting multiple ligands while retaining high specificity, thereby balancing therapeutic potency with the risk of tumor escape.

### 6.2 Combination therapy emerges as an imperative strategy

The immunosuppressive TME remains the principal barrier to the efficacy of NKG2D CAR-T cell therapy in solid tumors, positioning rationally designed combination regimens as a clinical necessity (2, 4, 45, 46).

Combination with PD-1/PD-L1 immune checkpoint inhibitors has become a pivotal strategy to enhance NKG2D CAR-T cell efficacy (42–44). PD-1 blockade significantly enhanced the anti-tumor efficacy of CAR-T cells against HCC, prolonging survival in treated mice by sustaining T cell effector function and reducing exhaustion (108). In gastric cancer, combining anti-PD-1 monoclonal antibodies with ULBP2 CAR-T cells not only prevented CAR-T cell exhaustion but also promoted central memory T-cell differentiation (58). Additionally, CAR-T cells engineered to secrete PD-1-blocking scFv provided localized, dual-mode checkpoint blockade through both autocrine and paracrine mechanisms, enhancing the activity of CAR-T cells and bystander tumor-specific T cells in clinically relevant syngeneic and xenogeneic PD-L1(+) hematologic and solid tumors (109). Notably, this strategy may offer an improved safety profile, as the secreted scFvs remain localized within the tumor, shielding CAR-T cells from PD-1-mediated inhibition while potentially avoiding toxicities linked to systemic checkpoint blockade. Mechanistically, PD-1 blockade disrupts PD-L1-induced SHP-2-dependent dephosphorylation of key signaling molecules (CD28, ZAP70), restoring IL-2 production, T cell proliferation, and central memory differentiation, and enhancing the persistence of NKG2D CAR-T cells within the immunosuppressive TME (110–113). PD-1 inhibition has also been shown to upregulate CXCR3 on CAR-T cells, facilitating their trafficking to tumor sites and boosting infiltration, proliferative capacity, and cytokine secretion (e.g., IFN-γ, TNF-α), thereby amplifying antitumor activity (114). Compensatory upregulation of alternative checkpoints (e.g., TIM-3, LAG-3) following PD-1 blockade may trigger immunosuppressive rebound, while systemic delivery of PD-1 inhibitors risks immune-related adverse events such as colitis, pneumonitis, dermatopathies, and thyroid dysfunction. Addressing these issues will require optimized dosing regimens, the development of multi-specific antibodies targeting complementary checkpoints, and localized delivery strategies to maximize efficacy while minimizing toxicity (115–118).

Furthermore, combining NKG2D CAR-T cells with TME-modulating agents such as MMP inhibitors offers a compelling strategy for solid tumor therapy. Soluble NKG2DLs (e.g., sMICA) arise from MMP-mediated cleavage of membrane-bound ligands, diminishing surface ligand density and impairing CAR-T recognition. MMP inhibitors such as SB-3CT effectively prevent ligand shedding, thereby restoring target availability and enhancing CAR-T cell functionality (36–39, 119, 120). In gastric cancer, MMP-specific inhibition has been shown to restore membrane-bound NKG2DL expression, heighten NK cell sensitivity to tumor cells, and suppress soluble ligand production-reversing key immune evasion pathways. However, indiscriminate MMP blockade can disrupt physiological tissue remodeling, causing toxicities such as musculoskeletal syndrome. Ongoing research is therefore focused on developing selective inhibitors targeting tumor-specific MMPs (e.g., MMP-2, MMP-9) to improve safety while maintaining therapeutic efficacy (121).

Moreover, combinatorial regimens incorporating agents that enhance tumor immunogenicity and inflammatory activation, such as radiotherapy, chemotherapy, epigenetic modulators, or immune potentiators, offer multifaceted therapeutic avenues and should prioritize boosting tumor immunogenicity beyond merely countering T cell exhaustion and ligand shedding. Combinatorial regimens that enhance tumor immunogenicity-such as DKK1 inhibition-mediated Wnt activation to upregulate NKG2DLs can prime or sustain CAR-T cell activity, establishing a paradigm for synergistic, pathway-targeted combination therapy (80). Physical and chemical interventions can upregulate NKG2DL expression, enhancing NKG2D CAR-T cell recognition and cytotoxicity. Radiotherapy triggers DNA damage and stress responses to increase surface NKG2DL density, while cisplatin selectively elevates NKG2DLs on malignant but not normal cells, boosting CAR-T efficacy with a favorable safety profile (61, 76). These insights drive a shift from monotherapy selection based solely on tumor morphology toward integrative regimens harnessing complementary mechanisms for synergistic benefit.

Epigenetic modulation to reverse transcriptional silencing of NKG2DLs represents another innovative avenue. The HDAC inhibitor VPA enhances histone acetylation to upregulate MICA/B surface expression, sensitizing tumor cells to immune attack without increasing soluble NKG2DLs. This strategy dismantles immune evasion at the transcriptional source but requires careful mitigation of off-target risks, including ligand induction in normal tissues and hematologic toxicity. Future efforts should focus on tumor-selective or locally delivered epigenetic agents, precision drug design, and tumor-type-specific mapping of NKG2DL silencing to enable personalized combinations (64, 100).

Combining immunomodulatory drugs to directly optimize CAR-T cell function represents a promising breakthrough strategy. For instance, lenalidomide-mediated degradation of the transcriptional repressors Ikaros (IKZF1) and Aiolos (IKZF3) relieves suppression of T cell activity, thereby activating the AP-1 transcription factor and ERK phosphorylation pathway to synergistically enhance CAR-T cell proliferative capacity and cytotoxic efficacy (79). This strategy of remodeling T cell intrinsic functionality to improve the TME provides a novel pathway to overcome immunosuppressive barriers in solid tumors. However, lenalidomide’s potential systemic inflammatory risks (e.g., CRS) necessitate rigorous clinical monitoring during translation, and the regulatory intricacies of its effects on downstream NKG2D signaling pathways require further elucidation to precisely optimize the therapeutic window.

### 6.3 Engineering enhancements to augment CAR-T cell functionality

To further optimize NKG2D CAR-T cells, future engineering strategies should focus on designing optimized structural configurations and equipping auxiliary functional modules. These approaches could either enhance the CAR-T cells intrinsic resistance to immunosuppressive TME or empower them to remodel the TME toward an immunostimulatory state. Multispecific CARs co-targeting NKG2D and TAAs have emerged as a mature engineering strategy for precision targeting. As organ-specific therapeutic targets continue to be discovered, this approach will persistently demonstrate transformative potential. Knockdown or knockout of NKG2DL expression in NKG2D CAR-T cells effectively prevents fratricide. Furthermore, silencing receptors that suppress immune cell function (e.g., PD-1, CTLA-4) through various methods maximizes effector cell potency. Soluble MICA (sMICA) potently inhibits NKG2D CAR-T cell function by competitively binding NKG2D and downregulating receptor expression. Future armored CAR-T cells engineered to secrete sMICA-neutralizing antibodies may address this challenge by neutralizing soluble ligands in the TME.

IL-15 is a potent cytokine that promotes T cell survival, proliferation, and infiltration into solid tumors (122–127). CAR-T cells engineered to express IL-15 exhibit enhanced persistence and effector function within hypoxic TME. In a study by Chen et al. NKG2D CAR-T cells engineered to express the IL-15/IL-15R complex (IL15C) demonstrated enhanced therapeutic efficacy against pancreatic cancer both *in vitro* and *in vivo (*88). However, IL-15 overexpression carries risks of uncontrolled T cell proliferation and CRS (128). Current research is exploring inducible expression systems such as those activated by tumor-specific promoters to mitigate these risks. The design strategies for armored CAR-T cells have sparked significant research interest. Future developments may not only enable cells to secrete natural factors but also incorporate artificial logic gates to enhance the functional versatility and specificity of secreted payloads. Beyond this, engineering receptor-ligand signaling pathways could potentially convert immunosuppressive factors in the TME into agonist functions upon membrane binding (87). Examples include engineering chimeric cytokine receptors (e.g., TGF-βRII/IL-21R) and targeted editing of specific receptors on CAR-T cells (129).

The engineering upgrades to NKG2D CAR-T cell structures fully leverage the advantages of biochemistry and molecular biology, illuminating novel pathways for solid tumor therapy from distinctive perspectives.

### 6.4 Safety and clinical translation

Although NKG2D CAR-T cell therapy has demonstrated encouraging antitumor activity in preclinical studies, this robust efficacy has not yet been consistently translated into durable clinical responses. This translational gap arises from multiple complex factors, including fundamental differences between experimental models and patient tumors, diverse immunosuppressive mechanisms within the TME, and intrinsic limitations of CAR-T cell products as injectable therapeutics.

#### 6.4.1 The gap between preclinical and clinical performance

Mouse xenograft models are unable to fully recapitulate the complex interactions between the human immune system and the TME. Even when significant tumor regression is observed in highly optimized immunodeficient mouse models, these systems fail to accurately reflect key features of human solid tumors, such as dense stromal barriers, immunosuppressive cell populations, and metabolic dysregulation (130). These factors contribute to insufficient CAR-T cell infiltration, progressive functional exhaustion, and limited persistence in patients. Moreover, preclinical studies often employ newly established tumors, whereas tumors in treatment-refractory patients are usually advanced, highly heterogeneous, and embedded within well-developed immunosuppressive microenvironments, rendering single-target strategies inadequate for durable clinical efficacy.

#### 6.4.2 In-depth analysis of safety risks

Cytokine-armored strategies (such as secretion of IL-7, or expression of dominant-negative TGFβ receptors) significantly enhance CAR-T cell infiltration and persistence in solid tumors (53, 131). However, these modifications simultaneously introduce substantial safety risks. Although cytokine-armored CAR-T cells can partially overcome local immunosuppression through sustained cytokine signaling, they may also trigger CRS via multiple interconnected mechanisms (132, 133). IL-7 and IL-15 secretion can directly drive excessive *in vivo* expansion and activation of CAR-T cells, resulting in massive proliferation and continuous release of pro-inflammatory cytokines, including IFN-γ and TNF-α, thereby initiating a cytokine cascade. In parallel, these cytokines activate innate immune cells such as macrophages and dendritic cells, which further secrete IL-6 and IL-8, forming a positive feedback loop that amplifies systemic inflammation. In a phase I clinical trial involving patients with metastatic castration-resistant prostate cancer, 38.5% of patients developed grade ≥2 CRS, including one fatal grade 4 event following lymphodepletion. Mechanistic analyses revealed that high-grade CRS was closely associated with excessive *in vivo* CAR-T cell expansion and markedly elevated inflammatory cytokine levels, while these patients simultaneously exhibited pronounced antitumor responses. These findings underscore the complex and delicate balance between therapeutic efficacy and toxicity risk in cytokine-armored CAR-T cell therapies (131). Additionally, cytokine-mediated activation of immune cells also increases vascular permeability, facilitating systemic dissemination of inflammatory mediators and further exacerbating CRS severity.

#### 6.4.3 Translational challenges and potential strategies

Multiple challenges continue to limit the effective translation of preclinical findings into clinical applications. First, preclinical models inadequately recapitulate the spatiotemporal heterogeneity of human TMEs. Although patient-derived organoids (PDOs) and patient-derived xenografts (PDXs) provide more clinically relevant systems, their high cost and limited throughput restrict widespread application. Second, preclinical studies typically use T cells from healthy donors, while patient-derived T cells often exhibit functional impairments due to tumor burden or prior treatments, which can impact the efficacy of CAR-T therapies.

Dose translation remains another critical challenge. Clinical trials have shown substantial inter-patient variability in CAR-T cell expansion kinetics, influenced by factors such as tumor burden, intensity of lymphodepletion, and the host’s immune status (51, 134). Additionally, the route of administration plays a crucial role in solid tumor treatment: intraperitoneal or intravenous delivery offers distinct distribution advantages across different tumor types, while CNS tumors face the additional barrier of the BBB.

Differences between preclinical and clinical studies are also evident in endpoint assessments. Animal studies typically prioritize tumor volume reduction and survival extension as primary endpoints, whereas clinical practice focuses more on disease control rate (DCR), progression-free survival (PFS), and patient quality of life. In patients with refractory solid tumors, NKG2D CAR-T therapy may provide disease stabilization and symptom relief rather than significant tumor shrinkage—benefits that are difficult to assess accurately in conventional mouse models.

Despite these challenges, the translational potential of NKG2D CAR-T cell therapy in solid tumors remains promising. Future research should focus on: developing more clinically predictive model systems; optimizing CAR structures and co-stimulatory domain combinations to balance efficacy and safety; incorporating multiple safety mechanisms such as logic-gated designs; and implementing personalized treatment strategies based on tumor NKG2D ligand expression profiles and shedding status to optimize dosing and administration protocols. Through multidisciplinary collaboration and precise risk assessment, NKG2D-targeted immunotherapy holds the potential to overcome current translational barriers and provide durable, safe treatment options for solid tumor patients.

## 7 Conclusion

Research on NKG2D CAR-T cell therapy for solid tumors has entered the era of combination regimens, with CAR-T cell immunotherapy integration emerging as the definitive future direction. Utilizing multimodal strategies to remodel the TME, enhance immune cell infiltration and functionality, and amplify therapeutic efficacy will constitute pivotal breakthroughs in this field.

## Glossary

The ADAM: A Disintegrin and Metalloproteinases
AP-1: Activator protein 1
BBB: Blood-Brain Barrier
Bcl-2: B-cell lymphocyte/leukemia 2 gene
B-NDG: NOD-Prkdcscid IL2rgtm1/Bcgen
CAF: Cancer-associated fibroblasts
CAR: Chimeric antigen receptor
CCL19: C-C Motif Chemokine Ligand 19
CIN: Cervical intraepithelial neoplasia
CNS: Central nervous system
CRS: Cytokine release syndrome
CTLA-4: Cytotoxic T-lymphocyte antigen 4
CX3CL1: C-X3-C motif chemokine ligand 1
CX3CR1: C-X3-C motif chemokine receptor 1
CXCR3: C-X-C motif chemokine receptor type 3
CXCR5: C-X-C motif chemokine receptor type 5
DAP10: DNAX-activating protein 10
DKK1: Dickkopf1
EPB41: Erythrocyte membrane protein band 4.1
ERK: Extracellular signal-regulated kinase
GBM: Glioblastoma
GM-CSF: Granulocyte-macrophage colony-stimulating factor
GPC3: Glypican-3
GPI: Glycosylphosphatidylinositol
GSCs: Glioblastoma stem cells
H3K27me3: Histone H3K27 trimethylation
HCC: Hepatocellular carcinoma
HDAC: Histone deacetylase
ICR: Inverted cytokine receptor
IDO: Indoleamine 2,3-dioxygenase
IKZF: Ikaros zinc finger
IL-15C: Chimeric IL-15 complex
LAG-3: Lymphocyte activation gene 3
M28z10: DAP10 intracellular signaling domain
MDSCs: Myeloid-derived suppressor cells
MHC: Major histocompatibility complex
MIC: Major Histocompatibility Complex Class I-Related Chain
MMP: Matrix Metalloproteinases
NKG2D: Natural killer group 2 member D
NKG2DLs: NKG2D Ligands
NSCLC: Non Small Cell Lung Cancer
PDAC: Pancreatic ductal adenocarcinomas
PDX: Patient-derived xenograft
SB-3CT: (4-Phenoxyphenylsulfonyl)methylthiirane
scFv: Single-Chain Fragment Variable
sMICA: Soluble form of MICA
TAAs: Tumor-Associated Antigens
TIGIT: T cell immunoreceptor with immunoglobulin and tyrosine-based inhibitory motif (ITIM) domain
TIM-3: T cell immunoglobulin and mucin-domain-containing-3
TME: Tumor microenvironment
TNBC: Triple Negative Breast Cancer
ULBP: UL16-binding protein
VPA: Valproic acid
αMUC1: Anti-MUC1
